# Chemical phases analysis of Barium in Ores by X-ray Fluorescence Spectroscopy

**DOI:** 10.1371/journal.pone.0326959

**Published:** 2025-07-11

**Authors:** Xiao Wang, Jiufen Liu, Boxin Feng, Xiaofeng Zhao, Ronghua Li, Liming Gan, Na Guo

**Affiliations:** 1 Xi’an Center of Mineral Resources Survey, China Geological Survey, Xi’an, China; 2 Technology Innovation Center for Gold Ore Exploration, China Geological Survey, Xi’an, China; 3 Command Center for Natural Resources Comprehensive Survey, China Geological Survey, Beijing, China; National Research Centre, EGYPT

## Abstract

Accurately determining the phase states of barium carbonate, barium silicate, and barium sulfate in ores. It’s crucial for advancing research on barium ore mineralization and improving beneficiation and smelting processes. This study aimed to investigate the integration of phase analysis and X-ray fluorescence spectrometry(XRF) to assess the phases of barium in ores, only requiring simple sample pretreatment before measurement. The acetic acid separation drip method was used for the determination of barium carbonate, while the hydrochloric acid separation drip method was used for barium silicate. Additionally, the fusion sample preparation method was applied for the analysis of barium sulfate. The results were consistent with those obtained using chemical methods, and the precision of the relative standard deviation(RSD) was less than or equal to 2.74%, satisfying the analytical requirements. This study combines chemical separation with XRF for continuous and precise phase determination. This approach enhances applicability of XRF in chemical phase analysis and provides a simpler, more environmentally friendly alternative to traditional techniques. It can be applied to barium phase analysis in general barium ores.

## Introduction

Barium has been widely used as a weighting agent for drilling operations, and as a conductive materials in various industries, such as metallurgy, petroleum, chemical, and mining [1–5]. In typical ores, barium mainly exists in three forms: carbonate, sulfate, and silicate. They are all significant barium-containing minerals. Barium carbonate and barium silicate have extensive applications in ceramics and electronics [6,7]. Barium sulfate primarily serves as a weighting agent in drilling mud for oil and gas extraction. It’s also a critical raw material for synthesizing barium-based chemical products [8–10]. Precisely determining the phase states of these minerals in ores is crucial for studying barium ore formation and developing subsequent beneficiation and smelting processes [8,11,12].

The analysis methods for chemical phases include the chemical method [13,14], X-ray fluorescence spectroscopy(XRF) [15,16], inductively coupled plasma atomic emission spectrometry (ICP-AES) and X-ray diffraction(XRD) [17–19]. XRF is used for composition analysis of substances [20–27]. It has the advantages of a wide detection range, high sensitivity and accuracy in analysis, and is easy to operate. It is suitable for the analysis of various solid and liquid materials [28–36]. In recent years, by combining extraction and other separation methods, XRF can be applied to the analysis of different trace elements [37–40]. It has been innovatively applied to the analysis of the chemical states of materials and the valence states [15,41]. In the conventional analysis of barium phase, chemical methods are mainly used, involving complex steps, long cycles, low efficiency, and difficulty in separation SrSO_4_, which affects the weighing of barium sulfate [42]. ICP-AES requires two high-temperature sample fusion steps and two filtration processes, which is intricate [43]. In contrast, XRF only necessitates sample fusion and preparation before direct instrumental analysis, enhancing efficiency and speed. Notably, for samples with high concentrations of barium sulfate, XRF demonstrates superior accuracy compared to other techniques [6]. Overall, the XRF method exhibits reduced interference, significantly decreases analysis time, and improves detection efficiency relative to other methods. For the analysis of barium phases in ores, Hou Wei et al. determined barium sulfate in the ore by preparing fusion samples [44]. Chen Jingwei et al. [6] used 10% acetic acid to isolate barium carbonate and added alumina to the original sample weight for subsequent analysis. Then, they determined barium sulfate through the melt sample preparation method. However, they didn’t consider the possible interference of barium silicate on the analytical results. This traditional method is mainly suitable for determining barium sulfate in simple barite compositions. Fan Zhiyu et al. used phase conversion partial pressure corrected headspace gas chromatography to determine barium sulfate in barite ore [7]. Although these methods are used to determine various barium ores, there are no reports on the instrumental determination methods for the continuous analysis of the chemical phases of barium compounds at present. Based on previous work, the present study investigated the application of XRF in barium phase analysis. In this study, XRF was used to continuously measure the three phases of barium carbonate, barium silicate, and barium sulfate in the ore. Up to now, no other literature has reported this particular result.

This paper describes improvements in the sequential separation and analysis of barium phases. Acetic acid isolates barium carbonate, while hydrochloric acid separates barium silicate. This method fulfills the phase analysis requirements for barium in common ores. Moreover, it incorporates XRF for rapid and accurate analysis, facilitating the transition from traditional chemical methods to instrumental techniques. In this way, we have successfully achieved the sequential separation of barium phases. It introduces XRF-based phase analysis for barium, offers both speed and accuracy. It can be promoted to conform to the trend towards instrumental analysis rather than traditional chemical methods.

## Materials and methods

### Main instruments and reagents

Instruments: An Axios X-ray fluorescence spectrometer (PANalytical, Amsterdam, The Netherlands) was used under the following operating conditions: a power of 4.0 kW, a maximum voltage of 60 kV, and a maximum current of 120 mA. The instrument is equipped with a rhodium target X-ray tube with a thin window (75 µm). Detailed instrument parameters for elemental analysis are presented in Table 1, and Table 2 provides information on the analysis of spectral lines and interference corrections.

**Table 1:** Sampling points of different soil column profiles in the study area.

Channel	Line	X-tal	Collimator(µm)	Detector	Tube filter	Angle(2θº)	Off set(2θº)	PHD1 LL	PHD2 UL
Rh1	KA-C	LiF 200	300	Hiper	None	18.4306	–	20	78
Ba	LA	LiF 200	300	Flow	None	87.1904	1.6198	33	71
Ti	KA	LiF 200	300	Flow	None	86.1514	−0.9288	29	71

**Table 2:** Matrix interference corrections for the analyzed elements.

Element	Analytical line	Interference line	Correction coefficient
Ba	LA	Ti	0.002173
Ti	KA	–	–

Intelligent high-frequency fusion sample equipment (domestic), Platinum crucible (wPt 95% + wAu 5%), Rigaku XRF specialized filter paper (30 mm), Sample holder (a circular plate made of organic glass, with a thickness of 8 mm and a diameter of 48 mm).

Silicon dioxide: The high-purity spectroscopic grade material was calcined at 1000 °C in a high- temperature furnace equipped with a silicon-carbon rod configuration for 1 hour.

Specialized XRF mixed flux composition: m(Li_2_B_4_O_7_):m(LiBO_2_):m(LiF) = 45:10:5, characterized by high purity. The mixture was accurately calcined at 650 °C in a high-temperature furnace with a silicon- carbon rod configuration before being used.

### Sample preparation

A powdered sample weighing precisely 0.3000 g (particle size less than 74 μm) was placed in a beaker. Subsequently, 10 ml of 5% acetic acid (v/v) was added, and the mixture was dissolved in a boiling bath for 30 minutes. After being removed from heat, the solution was allowed to cool and water was added to reach a final volume of 50 ml. The solution was then filtered using slow filter paper, ensuring that all of the solution and precipitate were transferred to the filter paper. Following filtration, the filter paper was washed 6–8 times with water, and the filtrate was made up to 100 mL in a volumetric flask. The Rigaku XRF specialized filter paper was suspended on a plastic ring, and 300 μL of the prepared solution was added to the filter paper using a pipette. The filter paper was subsequently allowed to air dry naturally in a dry room at room temperature. Afterward, it was placed on a sample holder for the determination of barium carbonate.

The precipitate enclosed in filter paper was transferred to a ceramic crucible. Then, the crucible was placed in a high-temperature furnace equipped with silicon-carbon rods. It was gradually heated to 450 °C, with the furnace door slightly open. After maintaining this temperature for 0.5 hours, the temperature was further raised to 650 °C. At this point, the furnace door was closed, and an additional 30 minutes of heat preservation was carried out. After being removed from the furnace, the content was transferred to a poly (trifluoroethylene) beaker. Next, 20 ml of 10% hydrochloric acid (v/v) was added to the beaker, and the mixture was dissolved in a boiling water bath for 30 minutes. Once it cooled down, the volume was adjusted to 25 ml with water. Then, the solution was filtered using slow filter paper. Along with the precipitate, the whole solution were transferred onto the filter paper. The filter paper was washed 6–8 times with water, and the filtrate was transferred to a volumetric flask and made up to 100 ml. Using a pipette, 300 μL of the above solution was added onto the filter paper. Subsequently, it was placed in a dry room at room temperature for natural air drying. Finally, it was put on a sample holder for the determination of barium silicate.

After separating the carbonate and silicate phases, carefully wrap the precipitate in filter paper and then transfer it to a ceramic crucible. Next, place this crucible in a high-temperature furnace equipped with a silicon carbon rod setup. Leave the furnace door slightly ajar. Gradually increase the temperature to 450 °C. After insulating for 30 minutes, the temperature was further increased to 650 °C. Then close the furnace door and let it insulate for an extra 30 minutes. Once removed from the furnace, let the crucible cool down. After it has cooled, weigh it and add silicon dioxide until the total weight reaches 0.3000 g. Add 6.0000 g of XRF specialized mixed flux to this mixture and transfer the whole content to a platinum-gold crucible. Then, use 6 drops of saturated lithium bromide and 3 drops of saturated ammonium nitrate. Place the crucible on a high-frequency fusion machine to ensure uniform melting. Pour the molten mixture into a preheated platinum-gold alloy mold. Let the solid formed cool in the air. Finally, put the cooled sample into an XRF fluorescence sample introduction device for further analysis.

## Results and discussion

### Carbonate phase separation condition selection

Low-concentration acetic acid solution can effectively dissolve barium carbonate in the ore under water bath heating. This forms soluble barium acetate, as shown in Eq (1). This method shows a low dissolution rate for barium silicate and barium sulfate, enabling the separation of barium carbonate and barium sulfate [45]. The study optimized the acetic acid concentration and dissolution time.


$$BaCO_{3}+2CH_{3}COOH\to Ba(CH_{3}COOH{)}_{2}+H_{2}O+CO_{2}\mathrm{\backslash ]}$$ (1)

**Fig 1:**
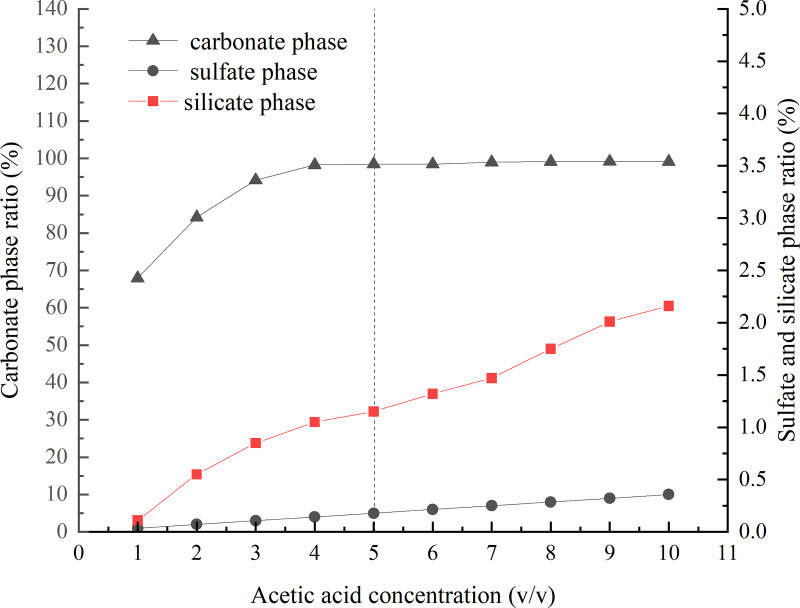
The results of carbonate, silicate and sulfate phases dissolution ratios at different acetic acid concentrations.

Different concentrations of acetic acid were tested for sample dissolution and the separation effect was determined by measuring the barium content in the filtrate. The results of the dissolution ratios of the carbonate, silicate, and sulfate phases at different concentrations of acetic acid are illustrated in Fig 1. It was revealed that at extremely low concentrations of acetic acid, the dissolution of the carbonate phase was limited. In contrast, with an increase in the concentration of acetic acid, there was a potential for the dissolution of some silicates. Therefore, a 5% (v/v) acetic acid solution was designated for the carbonate phase separation condition. For more information, see S1 Fig.

**Fig 2:**
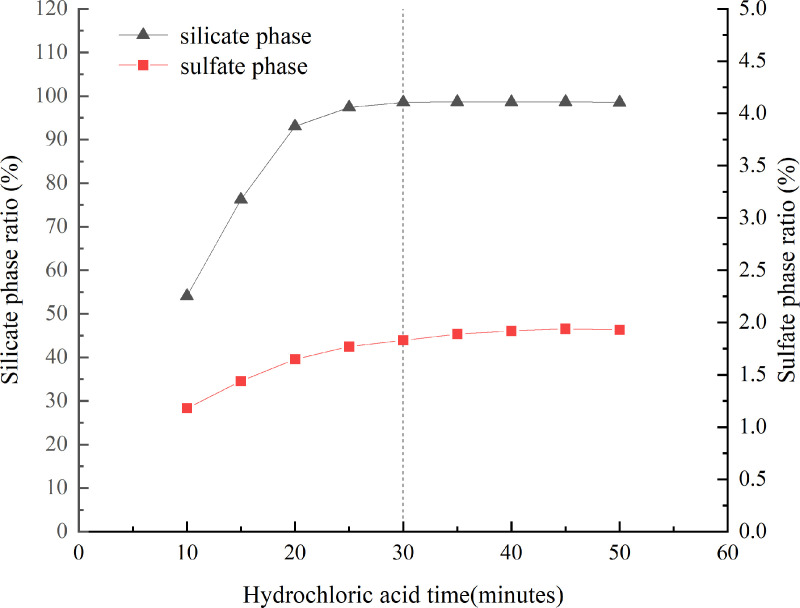
The results of carbonate, silicate and sulfate phases dissolution ratios at different time points.

Different times of acetic acid for sample dissolution were tested, and the separation effect was determined by measuring the barium content in the filtrate. The results of the witherite, barium feldspar, and barite dissolution ratios at different times are shown in Fig 2. The dissolution of the carbonate phase remained essentially unchanged when the dissolution time exceeded 20 minutes, and the optimal dissolution time was determined to be 30 minutes. For more information, see S2 Fig.

### Silicate phase separation condition

Under heated conditions, low-concentration hydrochloric acid can effectively dissolve barium silicate in ores, generating soluble barium acetate, as presented in Eq (2). This method shows a relatively low dissolution rate for barium sulfate, thereby enabling phase separation of barium silicate and barium sulfate. In this study, the concentration of hydrochloric acid and the dissolution time were optimized in a systematic way.


$${BaSiO}_{3}+2HCl\to {BaCl}_{2}+H_{2}{SiO}_{3}\mathrm{\backslash ]}$$ (2)

**Fig 3:**
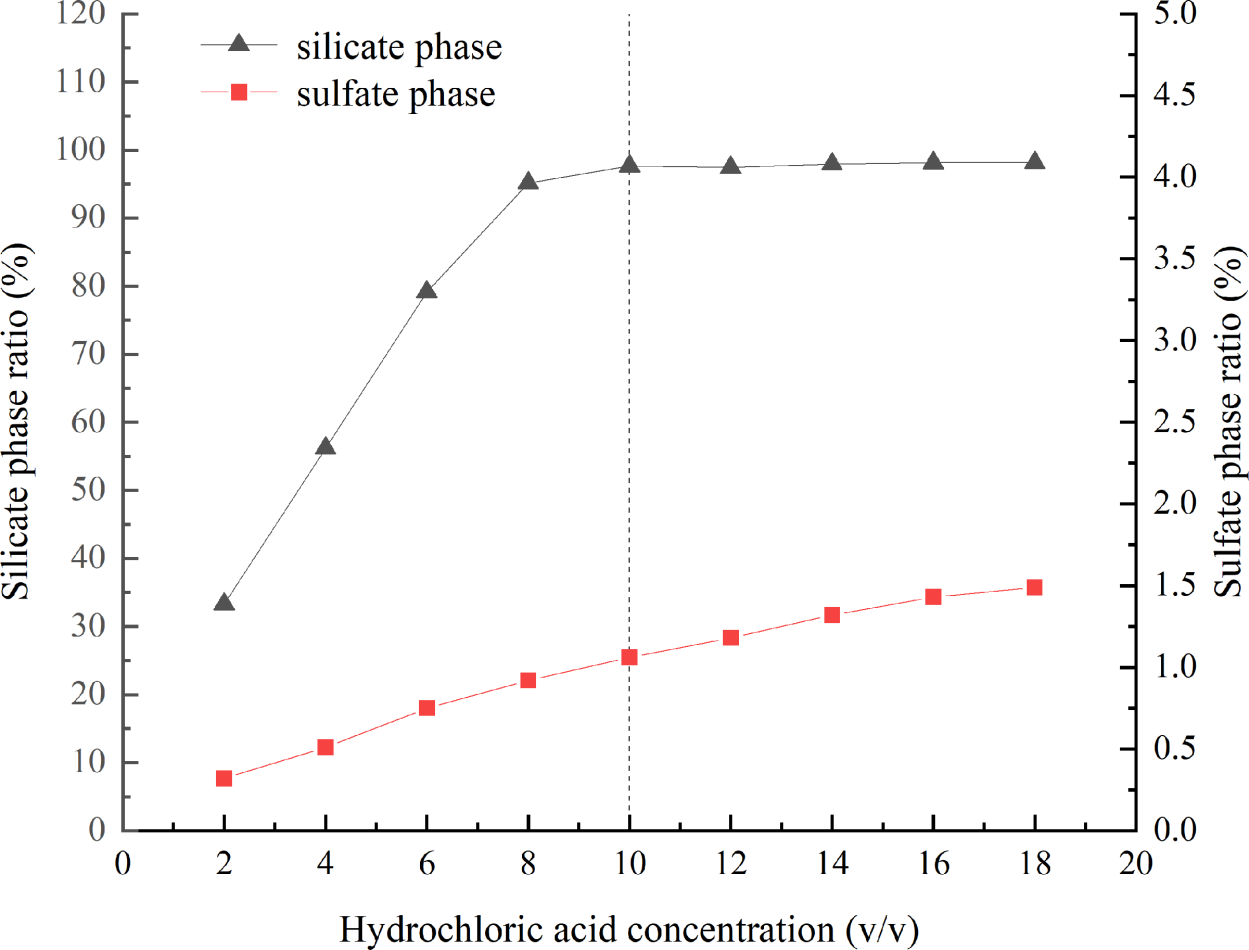
The results of silicate and sulfate phases dissolution ratios at different hydrochloric acid concentrations.

Different concentrations of hydrochloric acid were tested for sample dissolution, and the separation effect was determined by measuring the barium content in the filtrate. The results of the dissolution ratios of the silicate and sulfate phases at different hydrochloric acid concentrations are illustrated in Fig 3. It was found that at excessively low concentrations of hydrochloric acid, the dissolution of the silicate phase was limited. In contrast, with an increase in the concentration of acetic acid, there was the possibility that some barium sulfate might also dissolve (Fig 3). Therefore, a 10% (v/v) hydrochloric acid solution was nominated for the silicate phase separation condition. For more information, see S3 Fig.

**Fig 4:**
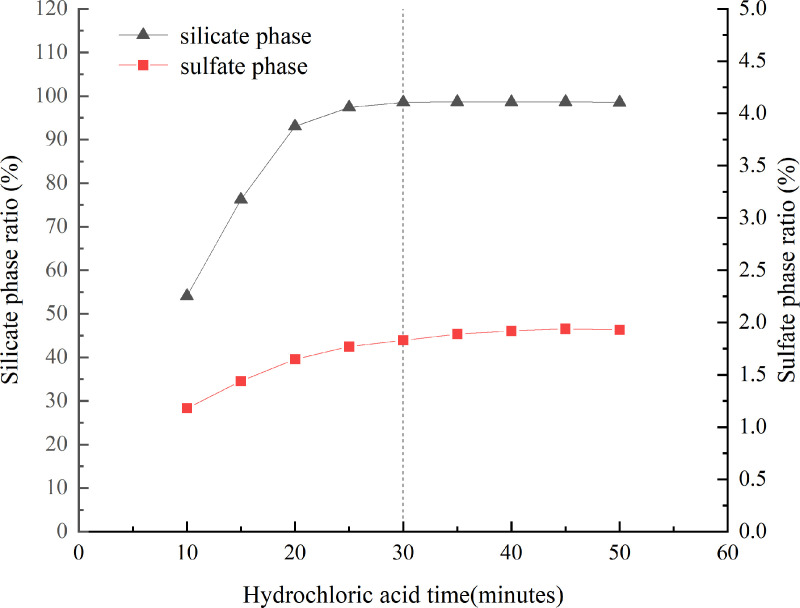
The results of silicate and sulfate phases dissolution ratios at different time point.

Different concentrations of hydrochloric acids were tested for sample dissolution, and the separation effect was determined by measuring the barium content in the filtrate. The dissolution ratios of the silicate and sulfate phases at various concentrations are illustrated in Fig 4. It was revealed that when the dissolution time exceeded 25 minutes, the dissolution of the silicate phase remained essentially unchanged. The optimal dissolution time was determined to be 30 minutes. For more information, see S4 Fig.

### Conditions for filter paper sample preparation

Experiments were conducted by dripping the sample onto a filter paper with a predetermined area of paraffin. This effectively limited the liquid sample to a fixed area. The filter paper was suspended and fixed using two aluminum clamps that could fully expand together. After natural air drying, the filter paper remained relatively flat, demonstrating promising repeatability [42]. By testing the diffusion radius of the sample on the filter paper with volumes ranging from 100 to 500 µL, it was determined that 300 µL was the optimal volume for drop-by-drop addition.

### Conditions for fusion sample preparation

Li_2_B_4_O_7_ is frequently utilized as a flux for fusing barium-bearing minerals. However, when it’s combined with heavy spar ores, the molten mixture becomes acidic and has a high viscosity, which leads to poor fusion. Through experiments, it was discovered that adding alkaline oxides can reduce the acidity of the molten mixture, decrease its viscosity, enhance the fusion effect, and improve the degree of vitrification. In this study, a mixed flux of Li_2_B_4_O_7_, LiBO_2_ and LiF (in a ratio of 45:10:5) was chosen. After the sample was separated and washed, it was weighed and silicon dioxide was added to restore the original mass. The mass of the mixed flux was 6.0000 g. Five glass slides were prepared with sample-to-flux dilution ratios of 1:10, 1:15, 1:20, 1:30, and 1:40 for testing. It was found that the dilution ratios of 1:20, 1:30 and 1:40 produced a fused material that flowing well. It was easy to remove from the mold and did not corrode the crucible, resulting in high quality fused samples (Table 3). Considering that higher dilution ratios could introduce larger measurement errors for samples with low concentrations, a dilution ratio of 1:20 was selected.

**Table 3:** The effects of sample dilution ratio on fusion efficiency.

Sample label	Dilution ratio	Fused materials	Fused sample detachment condition	Crucible corrosiveness
S-1	1 : 10	Poor Viscosity	Difficult	High
S-2	1 : 15	Average Viscosity	Difficult	Relatively Low
S-3	1 : 20	Good Viscosity	Easy	None
S-4	1 : 30	Excellent Viscosity	Easy	None
S-5	1 : 40	Excellent Viscosity	Easy	None

The experimental results indicated that the optimal melting temperature for the sample ranged from 1050 °C to 1150 °C, and the melting duration was over 8 minutes. Under these conditions, the fusion effect was quite obvious, and the measured results were stable. The optimal fusion parameters are presented in Table 4.

**Table 4:** Fusion disc preparation conditions.

Step	Oxidation	Ramp-up temperature	Hold	The primary oscillation	Stop	The secondary oscillation	Stop
Time (s)	60	90	120	180	5	180	10
Power (Hz)	65	72	83	–	–	–	–

### Establishment of standard curve

The standard curves of this method are divided into two categories. The first curve is made by filtering barium standard solution onto filter paper. It is used to determine barium carbonate and barium silicate. Its linear correlation coefficient (R) is 0.999. The second curve is prepared by melting barium ore standard material. It is used for determining the phase state of barium sulfate. Its linear correlation coefficient (R) is 0.998. To ensure the accuracy and stability of the measurement, corrections were made for the interference of TiKA spectral lines on BaLA spectral lines and for drift.

When using this method to determine barium, the BaLA line is chosen because of its high sensitivity. When analyzing barium carbonate, barium silicate, and barium sulfate phases, no filter is employed to keep the sensitivity. The detection limit is a bit higher than that of the ICP – AES method [3], yet it fully satisfies the requirements for determining the phase states of barium carbonate, barium silicate, and barium sulfate in ores.

### Precision of the developed method

A total of 7 barium ore samples were individually prepared using both filter paper and fusion disc methods. The Ba content was measured by an XRF spectrometer, and the average value along with the relative standard deviation (RSD) was calculated. The results are presented in Table 5. In particular, both drop sampling and fusion preparation methods exhibited RSDs for Ba determination less than 3%, which indicates excellent reproducibility.

**Table 5:** Precision testing of the method.

	Determination of Ba by 7 times (%)							Mean	RSD (%)
Carbonate	2.21	2.17	2.23	2.29	2.32	2.21	2.15	2.23	2.98
Silicate	1.11	1.07	1.03	1.09	1.02	0.99	1.05	1.06	3.29
Sulfate	40.41	40.11	40.23	40.32	40.19	40.55	40.49	40.33	0.40

### Accuracy of the developed method

The traditional approach involves dissolving barium carbonate with acetic acid and barium silicate with hydrochloric acid. After that, the filtrate is analyzed by ICP – AES. For the barium sulfate in the residue, it is first converted to barium carbonate by high – temperature fusion with sodium carbonate. Then, it is dissolved in hydrochloric acid. Next, sulfuric acid is used to precipitate it as barium sulfate to separate it from impurities. Finally, the content of barium sulfate is determined through gravimetric analysis.

By comparing and validating against traditional chemical methods, this paper conducts a systematic investigation into the phase characteristics of barium carbonate, barium silicate, and barium sulfate of 3 ore samples. The findings indicate that the accuracy of this method is highly consistent with the results obtained from traditional chemical analysis techniques (Table 6). This method simplifies the operational process. It effectively avoids complex steps such as impurity removal, which are typical of the traditional gravimetric method. Additionally, it eliminates repetitive procedures like fusion and filtration separation, thereby enhancing detection efficiency by 3 to 5 times.

**Table 6:** Results and RSDs of various methods for determining three samples.

Phase	Sample 1		Sample 2		Sample 3	
	Proposed method	Chemical method	Proposed method	Chemical method	Proposed method	Chemical method
Carbonate (%)	2.19	2.17	3.26	3.29	9.54	9.56
Silicate (%)	1.07	1.03	0.96	1.01	1.23	1.25
Sulfate (%)	40.35	40.39	93.43	93.47	72.17	72.23

Method 1 denotes the approach utilized in this article, whereas Method 2 represents the conventional chemical method.

## Conclusions

**Fig 5:**
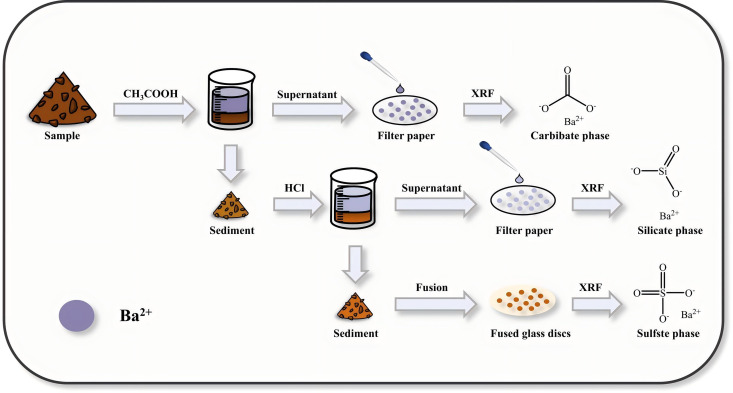
Flowchart. The technical flow chart of the determination of barium in different phases by XRFS method is presented by rapid separation of carbonate and silicate phases, combined with the preparation method of drop sample and fusion disc.

The main advantage of this study is its capacity to continuously determine three phases in barium ore. This is achieved by integrating phase analysis with XRF. A brand – new technique for continuously determining various barium phases using XRF has been developed. This technique includes quickly separating the carbonate and silicate phases, along with fusion plate preparation methods (as shown in Fig 5). This method is not only suitable for the phase analysis of barium in standard ores, but it also has the benefits of high speed, high accuracy, and great reproducibility. As a result, it is a practical alternative to traditional chemical methods.

## Supporting information

S1 Fig The results of carbonate, silicate and sulfate phases dissolution ratios at different acetic acid concentrations. (XLSX)

S2 Fig The results of carbonate, silicate and sulfate phases dissolution ratios at different time points. (XLSX)

S3 Fig The results of silicate and sulfate phases dissolution ratios at different hydrochloric acid concentrations. (XLSX)

S4 Fig The results of silicate and sulfate phases dissolution ratios at different time point. (XLSX)
